# *N*-(3-Nitro­phen­yl)maleamic acid

**DOI:** 10.1107/S1600536810022245

**Published:** 2010-06-16

**Authors:** B. Thimme Gowda, Miroslav Tokarčík, K. Shakuntala, Jozef Kožíšek, Hartmut Fuess

**Affiliations:** a Department of Chemistry, Mangalore University, Mangalagangotri 574 199, Mangalore, India; b Faculty of Chemical and Food Technology, Slovak Technical University, Radlinského 9, SK-812 37 Bratislava, Slovak Republic; c Institute of Materials Science, Darmstadt University of Technology, Petersenstrasse 23, D-64287 Darmstadt, Germany

## Abstract

In the title compound, C_10_H_8_N_2_O_5_, the mol­ecule is slightly distorted from planarity. The mol­ecular structure is stabilized by two intra­molecular hydrogen bonds. The first is a short O—H⋯O hydrogen bond (H⋯O distance = 1.57 Å) within the maleamic acid unit and the second is a C—H⋯O hydrogen bond (H⋯O distance = 2.24 Å) which connects the amide group with the benzene ring. The nitro group is twisted by 6.2 (2)° out of the plane of the benzene ring. The crystal structure manifests a variety of hydrogen bonding. The packing is dominated by a strong inter­molecular N—H⋯O inter­action which links the mol­ecules into chains running along the *b* axis. The chains within a plane are further assembled by three additional types of inter­molecular C—H⋯O hydrogen bonds to form a sheet parallel to the (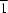
01) plane.

## Related literature

For studies on the effect of ring- and side-chain substitutions on the crystal structures of amides, see: Gowda, Tokarčík, Kožíšek *et al.* (2010 ▶); Gowda *et al.* (2010*a* ▶,*b* ▶); Prasad *et al.* (2002 ▶). For hydrogen-bond motifs, see: Bernstein *et al.* (1995 ▶).
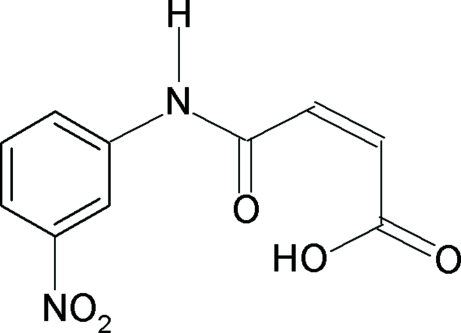


## Experimental

### 

#### Crystal data

C_10_H_8_N_2_O_5_
*M* *_r_* = 236.18
Monoclinic, 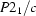

*a* = 7.9965 (2) Å
*b* = 14.0253 (3) Å
*c* = 9.1026 (2) Å
β = 100.147 (3)°
*V* = 1004.92 (4) Å^3^
*Z* = 4
Mo *K*α radiation
μ = 0.13 mm^−1^
*T* = 295 K
0.57 × 0.33 × 0.28 mm

#### Data collection

Oxford Diffraction Gemini R CCD diffractometer
Absorption correction: analytical (*CrysAlis PRO*; Oxford Diffraction, 2009 ▶) *T*_min_ = 0.926, *T*_max_ = 0.971
17136 measured reflections
1793 independent reflections
1544 reflections with *I* > 2σ(*I*)
*R*_int_ = 0.023

#### Refinement

*R*[*F* ^2^ > 2σ(*F* ^2^)] = 0.034
*wR*(*F* ^2^) = 0.097
*S* = 1.08
1793 reflections
154 parameters
H-atom parameters constrained
Δρ_max_ = 0.15 e Å^−3^
Δρ_min_ = −0.19 e Å^−3^

### 

Data collection: *CrysAlis PRO* (Oxford Diffraction, 2009 ▶); cell refinement: *CrysAlis PRO*; data reduction: *CrysAlis PRO*; program(s) used to solve structure: *SHELXS97* (Sheldrick, 2008 ▶); program(s) used to refine structure: *SHELXL97* (Sheldrick, 2008 ▶); molecular graphics: *ORTEP-3* (Farrugia, 1997 ▶) and *DIAMOND* (Brandenburg, 2002 ▶); software used to prepare material for publication: *SHELXL97*, *PLATON* (Spek, 2009 ▶) and *WinGX* (Farrugia, 1999 ▶).

## Supplementary Material

Crystal structure: contains datablocks I, global. DOI: 10.1107/S1600536810022245/ds2039sup1.cif

Structure factors: contains datablocks I. DOI: 10.1107/S1600536810022245/ds2039Isup2.hkl

Additional supplementary materials:  crystallographic information; 3D view; checkCIF report

## Figures and Tables

**Table 1:** Hydrogen-bond geometry (Å, °)

*D*—H⋯*A*	*D*—H	H⋯*A*	*D*⋯*A*	*D*—H⋯*A*
O2—H2*A*⋯O1	0.93	1.57	2.4978 (13)	176
C6—H6⋯O1	0.93	2.24	2.8302 (15)	121
N1—H1*N*⋯O3^i^	0.86	2.05	2.8929 (14)	167
C10—H10⋯O3^i^	0.93	2.51	3.2781 (17)	140
C3—H3⋯O5^ii^	0.93	2.57	3.2959 (17)	135
C9—H9⋯O4^iii^	0.93	2.51	3.1793 (17)	129
C8—H8⋯O2^iii^	0.93	2.57	3.4877 (17)	170

Symmetry codes: (i) 
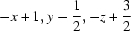
; (ii) 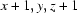; (iii) 
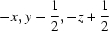
.

**Fig. 1.**
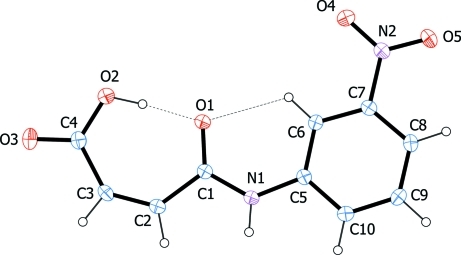
Molecular structure of (I) showing the atom labelling scheme. Displacement ellipsoids are drawn at the 30% probability level. Two short intramolecular bonds are indicated by dashed lines. H atoms are represented as small spheres of arbitrary radii.

**Fig. 2.**
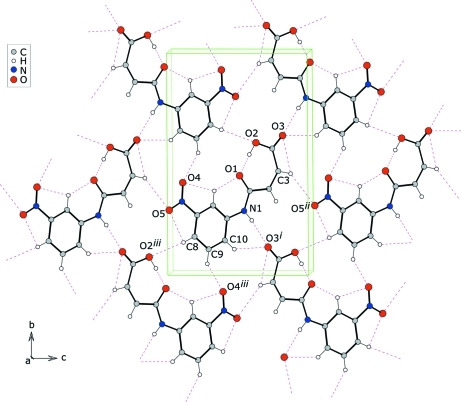
Part of crystal structure of (I) viewed down the a axis and showing a two-dimensional network of molecules linked by several types of intermolecular N–H···O and C–H···O hydrogen bonds (dashed lines). Symmetry codes (i): -x + 1, y - 1/2, -z + 3/2; (ii): x + 1, y, z + 1; (iii): -x, y - 1/2, -z + 1/2.

## supplementary crystallographic information

### Comment

In the present study, as a part of studying the effect of ring and side chain substitutions on the crystal structures of biologically significant amides (Gowda *et al.*, 2010*a,b,c*; Prasad *et al.*, 2002), the crystal structure of *N*-(3-nitrophenyl)maleamic acid (I) has been determined (Fig. 1). The conformation of the N—H in the amide segment is *anti* to the C=O bond and is also *anti* to the *meta*-nitro group in the phenyl ring.

In the maleamic acid moiety, the amide C=O bond is *anti* to the adjacent C—H bond, while the carboxyl C=O bond is *syn* to the adjacent C—H bond. The observed rare *anti* conformation of the C=O and O—H bonds of the acid group is similar to that obsrved in *N*-(2-methylphenyl)-maleamic acid (Gowda *et al.*, 2010*b*), *N*-(3-chlorophenyl)-maleamic acid (Gowda *et al.*, 2010*c*) and *N*-(3,5-dichlorophenyl)- maleamic acid (Gowda *et al.*, 2010*a*).

The molecule in (I) is slightly distorted from planarity as indicated by the dihedral angle of 4.5 (1)° between the least squares planes of the maleamic acid unit (r.m.s. deviation of 0.050 Å) and the phenyl ring. The molecular structure (Fig. 1) is stabilized by two intramolecular hydrogen bonds (Table 1). The first is a short O–H···O hydrogen bond ((H···O distance of 1.57 Å) within the maleamic acid unit; the second one is a C–H···O hydrogen bond (H···O distance of 2.24 Å) which connects the amide group with the phenyl ring. The nitro group - known to be a strong electron- withdrawing substituent - opens up the *ipso* C–C–C angle and narrows the two adjacent intracyclic angles. This fact is evident from the intracyclic bond angles C6–C7–C8, C5–C6–C7 and C7–C8–C9 of 123.99 (12)°, 117.49 (12)° and 117.64 (12)° respectively. The nitro group is twisted 6.2 (2)° out of the plane of the phenyl ring.

The crystal structure (Fig. 2) manifests a variety of hydrogen bonding. The packing is dominated by a strong intermolecular N–H···O interaction (H···O distance of 2.05 Å) which links the molecules into the chains running along the *b* axis. The chains within a plane are further assembled by additional three types of intermolecular C–H···O hydrogen bonds to form a sheet parallel to the (-1 0 1) plane (Bernstein *et al.*, 1995).

### Experimental

The solution of maleic anhydride (0.025 mol) in toluene (25 ml) was treated dropwise with the solution of 3-nitroaniline (0.025 mol) also in toluene (20 ml) with constant stirring. The resulting mixture was warmed with stirring for over 30 min and set aside for an additional 30 min at room temperature for completion of the reaction. The mixture was then treated with dilute hydrochloric acid to remove the unreacted 3-nitroaniline. The resultant solid *N*-(3-nitrophenyl)maleamic acid was filtered under suction and washed thoroughly with water to remove the unreacted maleic anhydride and maleic acid. It was recrystallized to constant melting point from ethanol. The purity of the compound was checked by elemental analysis and characterized by its infrared spectra.

Prism like light brown single crystals used in X-ray diffraction studies were grown in an ethanol solution by slow evaporation at room temperature.

### Refinement

All H atoms were visible in difference maps. The positions of carboxyl and amide H atoms were tested in preliminary refinement using a soft restraints on the O–H and N–H distances. Finally, all H atoms were positioned with idealized geometry using a riding model with the distances C–H = 0.93 Å, N–H = 0.86 Å and O–H = 0.93 Å. The *U*_iso_(H) values were set at 1.2*U*_eq_(C aromatic, N) and 1.5*U*_eq_(O).

### Crystal data

**Table:**

C_10_H_8_N_2_O_5_	*F*(000) = 488
*M**_r_* = 236.18	*D*_x_ = 1.561 Mg m^−^^3^
Monoclinic, *P*2_1_/*c*	Mo *K*α radiation, λ = 0.71073 Å
Hall symbol: -P 2ybc	Cell parameters from 10218 reflections
*a* = 7.9965 (2) Å	θ = 2.3–29.4°
*b* = 14.0253 (3) Å	µ = 0.13 mm^−^^1^
*c* = 9.1026 (2) Å	*T* = 295 K
β = 100.147 (3)°	Prism, light brown
*V* = 1004.92 (4) Å^3^	0.57 × 0.33 × 0.28 mm
*Z* = 4	

### Data collection

**Table:**

Oxford Diffraction Gemini R CCD diffractometer	1793 independent reflections
graphite	1544 reflections with *I* > 2σ(*I*)
Detector resolution: 10.434 pixels mm^-1^	*R*_int_ = 0.023
ω scans	θ_max_ = 25.1°, θ_min_ = 2.6°
Absorption correction: analytical (*CrysAlis PRO*; Oxford Diffraction, 2009)	*h* = −9→9
*T*_min_ = 0.926, *T*_max_ = 0.971	*k* = −16→16
17136 measured reflections	*l* = −10→10

### Refinement

**Table:**

Refinement on *F*^2^	Primary atom site location: structure-invariant direct methods
Least-squares matrix: full	Secondary atom site location: difference Fourier map
*R*[*F*^2^ > 2σ(*F*^2^)] = 0.034	Hydrogen site location: inferred from neighbouring sites
*wR*(*F*^2^) = 0.097	H-atom parameters constrained
*S* = 1.08	*w* = 1/[σ^2^(*F*_o_^2^) + (0.0618*P*)^2^ + 0.1002*P*] where *P* = (*F*_o_^2^ + 2*F*_c_^2^)/3
1793 reflections	(Δ/σ)_max_ < 0.001
154 parameters	Δρ_max_ = 0.15 e Å^−^^3^
0 restraints	Δρ_min_ = −0.19 e Å^−^^3^

### Special details

**Table:**

Geometry. All e.s.d.'s (except the e.s.d. in the dihedral angle between two l.s. planes) are estimated using the full covariance matrix. The cell e.s.d.'s are taken into account individually in the estimation of e.s.d.'s in distances, angles and torsion angles; correlations between e.s.d.'s in cell parameters are only used when they are defined by crystal symmetry. An approximate (isotropic) treatment of cell e.s.d.'s is used for estimating e.s.d.'s involving l.s. planes.
Refinement. Refinement of *F*^2^ against ALL reflections. The weighted *R*-factor *wR* and goodness of fit *S* are based on *F*^2^, conventional *R*-factors *R* are based on *F*, with *F* set to zero for negative *F*^2^. The threshold expression of *F*^2^ > σ(*F*^2^) is used only for calculating *R*-factors(gt) *etc*. and is not relevant to the choice of reflections for refinement. *R*-factors based on *F*^2^ are statistically about twice as large as those based on *F*, and *R*- factors based on ALL data will be even larger.

### Fractional atomic coordinates and isotropic or equivalent isotropic displacement parameters (Å^2^)

**Table:**

	*x*	*y*	*z*	*U*_iso_*/*U*_eq_	
C1	0.30465 (16)	0.37499 (8)	0.57539 (15)	0.0361 (3)	
C2	0.44044 (17)	0.38197 (9)	0.70806 (15)	0.0406 (3)	
H2	0.4755	0.3245	0.7543	0.049*	
C3	0.51952 (16)	0.45956 (10)	0.77048 (15)	0.0418 (3)	
H3	0.5991	0.4471	0.8559	0.05*	
C4	0.50454 (17)	0.56183 (10)	0.72945 (15)	0.0421 (3)	
C5	0.13141 (15)	0.25342 (9)	0.41799 (14)	0.0342 (3)	
C6	0.02666 (15)	0.31456 (9)	0.32224 (14)	0.0365 (3)	
H6	0.0378	0.3804	0.3312	0.044*	
C7	−0.09413 (16)	0.27380 (9)	0.21369 (13)	0.0357 (3)	
C8	−0.11652 (17)	0.17731 (9)	0.19464 (16)	0.0422 (3)	
H8	−0.1995	0.1528	0.1196	0.051*	
C9	−0.01165 (19)	0.11799 (9)	0.29057 (17)	0.0476 (4)	
H9	−0.0236	0.0522	0.2806	0.057*	
C10	0.11119 (17)	0.15542 (9)	0.40145 (15)	0.0417 (3)	
H10	0.181	0.1146	0.4656	0.05*	
N1	0.25906 (14)	0.28534 (7)	0.53544 (12)	0.0389 (3)	
H1N	0.3151	0.2413	0.5885	0.047*	
N2	−0.20553 (14)	0.33698 (8)	0.11149 (12)	0.0442 (3)	
O1	0.23643 (13)	0.44532 (6)	0.50694 (11)	0.0487 (3)	
O2	0.38964 (13)	0.59058 (7)	0.61814 (12)	0.0547 (3)	
H2A	0.3292	0.5383	0.5739	0.082*	
O3	0.60038 (15)	0.61868 (8)	0.80038 (13)	0.0649 (3)	
O4	−0.17942 (14)	0.42253 (7)	0.11665 (12)	0.0596 (3)	
O5	−0.32118 (15)	0.30062 (8)	0.02350 (14)	0.0752 (4)	

### Atomic displacement parameters (Å^2^)

**Table:**

	*U*^11^	*U*^22^	*U*^33^	*U*^12^	*U*^13^	*U*^23^
C1	0.0377 (7)	0.0306 (7)	0.0363 (7)	0.0005 (5)	−0.0039 (6)	0.0012 (5)
C2	0.0439 (7)	0.0332 (7)	0.0393 (7)	0.0036 (5)	−0.0078 (6)	0.0031 (5)
C3	0.0418 (7)	0.0415 (8)	0.0355 (7)	0.0014 (5)	−0.0115 (6)	−0.0003 (6)
C4	0.0467 (8)	0.0376 (7)	0.0381 (7)	−0.0022 (6)	−0.0034 (6)	−0.0044 (6)
C5	0.0359 (7)	0.0313 (7)	0.0326 (7)	−0.0003 (5)	−0.0018 (5)	−0.0013 (5)
C6	0.0406 (7)	0.0278 (6)	0.0373 (7)	−0.0016 (5)	−0.0035 (6)	−0.0011 (5)
C7	0.0372 (7)	0.0336 (7)	0.0334 (7)	0.0013 (5)	−0.0014 (5)	0.0014 (5)
C8	0.0431 (7)	0.0354 (7)	0.0429 (7)	−0.0039 (5)	−0.0069 (6)	−0.0052 (5)
C9	0.0559 (9)	0.0260 (7)	0.0547 (9)	−0.0017 (6)	−0.0071 (7)	−0.0031 (6)
C10	0.0452 (7)	0.0310 (7)	0.0441 (7)	0.0027 (5)	−0.0050 (6)	0.0023 (6)
N1	0.0424 (6)	0.0290 (5)	0.0388 (6)	0.0021 (4)	−0.0106 (5)	0.0024 (4)
N2	0.0475 (7)	0.0373 (7)	0.0413 (6)	0.0004 (5)	−0.0100 (5)	0.0006 (5)
O1	0.0556 (6)	0.0307 (5)	0.0493 (6)	0.0003 (4)	−0.0201 (5)	0.0031 (4)
O2	0.0659 (7)	0.0326 (6)	0.0549 (6)	−0.0029 (4)	−0.0190 (5)	0.0035 (4)
O3	0.0759 (8)	0.0444 (6)	0.0630 (7)	−0.0147 (5)	−0.0195 (6)	−0.0104 (5)
O4	0.0713 (7)	0.0326 (6)	0.0637 (7)	−0.0011 (5)	−0.0194 (5)	0.0051 (5)
O5	0.0769 (8)	0.0490 (7)	0.0778 (8)	−0.0031 (6)	−0.0463 (7)	0.0003 (6)

### Geometric parameters (Å, °)

**Table:**

C1—O1	1.2406 (15)	C6—H6	0.93
C1—N1	1.3414 (16)	C7—C8	1.3721 (18)
C1—C2	1.4782 (19)	C7—N2	1.4670 (16)
C2—C3	1.3343 (19)	C8—C9	1.378 (2)
C2—H2	0.93	C8—H8	0.93
C3—C4	1.4817 (19)	C9—C10	1.3820 (19)
C3—H3	0.93	C9—H9	0.93
C4—O3	1.2106 (17)	C10—H10	0.93
C4—O2	1.3059 (17)	N1—H1N	0.86
C5—C10	1.3890 (18)	N2—O4	1.2174 (15)
C5—C6	1.3925 (17)	N2—O5	1.2231 (15)
C5—N1	1.4145 (16)	O2—H2A	0.93
C6—C7	1.3784 (17)		
			
O1—C1—N1	122.32 (12)	C8—C7—N2	117.68 (11)
O1—C1—C2	123.53 (11)	C6—C7—N2	118.33 (11)
N1—C1—C2	114.14 (10)	C7—C8—C9	117.64 (12)
C3—C2—C1	128.80 (12)	C7—C8—H8	121.2
C3—C2—H2	115.6	C9—C8—H8	121.2
C1—C2—H2	115.6	C8—C9—C10	120.55 (12)
C2—C3—C4	132.14 (13)	C8—C9—H9	119.7
C2—C3—H3	113.9	C10—C9—H9	119.7
C4—C3—H3	113.9	C9—C10—C5	120.61 (12)
O3—C4—O2	120.21 (13)	C9—C10—H10	119.7
O3—C4—C3	119.18 (13)	C5—C10—H10	119.7
O2—C4—C3	120.60 (12)	C1—N1—C5	128.83 (11)
C10—C5—C6	119.72 (12)	C1—N1—H1N	115.6
C10—C5—N1	116.73 (11)	C5—N1—H1N	115.6
C6—C5—N1	123.54 (11)	O4—N2—O5	122.75 (11)
C7—C6—C5	117.49 (12)	O4—N2—C7	119.35 (10)
C7—C6—H6	121.3	O5—N2—C7	117.90 (11)
C5—C6—H6	121.3	C4—O2—H2A	109.5
C8—C7—C6	123.99 (12)		
			
O1—C1—C2—C3	−4.7 (2)	C8—C9—C10—C5	−0.1 (2)
N1—C1—C2—C3	176.01 (13)	C6—C5—C10—C9	0.2 (2)
C1—C2—C3—C4	−1.9 (3)	N1—C5—C10—C9	179.35 (12)
C2—C3—C4—O3	−175.18 (15)	O1—C1—N1—C5	−1.3 (2)
C2—C3—C4—O2	4.8 (2)	C2—C1—N1—C5	177.97 (11)
C10—C5—C6—C7	−0.04 (18)	C10—C5—N1—C1	179.91 (12)
N1—C5—C6—C7	−179.15 (11)	C6—C5—N1—C1	−1.0 (2)
C5—C6—C7—C8	−0.15 (19)	C8—C7—N2—O4	−173.53 (12)
C5—C6—C7—N2	−179.87 (11)	C6—C7—N2—O4	6.21 (18)
C6—C7—C8—C9	0.2 (2)	C8—C7—N2—O5	6.09 (18)
N2—C7—C8—C9	179.91 (12)	C6—C7—N2—O5	−174.17 (12)
C7—C8—C9—C10	0.0 (2)		

### Hydrogen-bond geometry (Å, °)

**Table:**

*D*—H···*A*	*D*—H	H···*A*	*D*···*A*	*D*—H···*A*
O2—H2A···O1	0.93	1.57	2.4978 (13)	176
C6—H6···O1	0.93	2.24	2.8302 (15)	121
N1—H1N···O3^i^	0.86	2.05	2.8929 (14)	167
C10—H10···O3^i^	0.93	2.51	3.2781 (17)	140
C3—H3···O5^ii^	0.93	2.57	3.2959 (17)	135
C9—H9···O4^iii^	0.93	2.51	3.1793 (17)	129
C8—H8···O2^iii^	0.93	2.57	3.4877 (17)	170

Symmetry codes: (i) −*x*+1, *y*−1/2, −*z*+3/2; (ii) *x*+1, *y*, *z*+1; (iii) −*x*, *y*−1/2, −*z*+1/2.

## References

[bb1] Bernstein, J., Davis, R. E., Shimoni, L. & Chang, N.-L. (1995). *Angew. Chem. Int. Ed. Engl.* **34**, 1555–1573.

[bb2] Brandenburg, K. (2002). *DIAMOND* Crystal Impact GbR, Bonn, Germany.

[bb3] Farrugia, L. J. (1997). *J. Appl. Cryst.* **30**, 565.

[bb4] Farrugia, L. J. (1999). *J. Appl. Cryst.* **32**, 837–838.

[bb5] Gowda, B. T., Tokarčík, M., Kožíšek, J., Shakuntala, K. & Fuess, H. (2010). *Acta Cryst.* E**66**, o51. 10.1107/S1600536809051484 PMC2980102 21580154

[bb6] Gowda, B. T., Tokarčík, M., Shakuntala, K., Kožíšek, J. & Fuess, H. (2010*a*). *Acta Cryst.* E**66**, o1554. 10.1107/S160053681002012X PMC3006921 21587799

[bb7] Gowda, B. T., Tokarčík, M., Shakuntala, K., Kožíšek, J. & Fuess, H. (2010*b*). *Acta Cryst.* E**66**, o1643. 10.1107/S1600536810021446 PMC3006958 21587872

[bb8] Oxford Diffraction (2009). *CrysAlis PRO* Oxford Diffraction Ltd, Abingdon, Oxfordshire, England.

[bb9] Prasad, S. M., Sinha, R. B. P., Mandal, D. K. & Rani, A. (2002). *Acta Cryst.* E**58**, o1296–o1297.

[bb10] Sheldrick, G. M. (2008). *Acta Cryst.* A**64**, 112–122. 10.1107/S0108767307043930 18156677

[bb11] Spek, A. L. (2009). *Acta Cryst.* D**65**, 148–155. 10.1107/S090744490804362X PMC2631630 19171970

